# Racial Discrimination, Neural Connectivity, and Epigenetic Aging Among Black Women

**DOI:** 10.1001/jamanetworkopen.2024.16588

**Published:** 2024-06-13

**Authors:** Aziz Elbasheir, Seyma Katrinli, Breanne E. Kearney, Ruth A. Lanius, Nathaniel G. Harnett, Sierra E. Carter, Timothy D. Ely, Bekh Bradley, Charles F. Gillespie, Jennifer S. Stevens, Adriana Lori, Sanne J. H. van Rooij, Abigail Powers, Tanja Jovanovic, Alicia K. Smith, Negar Fani

**Affiliations:** 1 Department of Psychiatry and Behavioral Sciences, Emory University School of Medicine, Atlanta, Georgia; 2 Department of Neuroscience, Schulich School of Medicine and Dentistry, Western University, London, Ontario, Canada; 3 Department of Psychiatry, Schulich School of Medicine and Dentistry, Western University, London, Ontario, Canada; 4 Division of Depression and Anxiety, McLean Hospital, Belmont, Massachusetts; 5 Department of Psychiatry, Harvard Medical School, Boston, Massachusetts; 6 Department of Psychology, Georgia State University, Atlanta; 7 Atlanta Veterans Affairs Medical Center, Atlanta, Georgia; 8 Department of Psychiatry and Behavioral Neurosciences, Wayne State University, Detroit, Michigan; 9 Department of Gynecology and Obstetrics, Emory University School of Medicine, Atlanta, Georgia

## Abstract

**Importance:**

Racial discrimination increases the risk of adverse brain health outcomes, potentially via neuroplastic changes in emotion processing networks. The involvement of deep brain regions (brainstem and midbrain) in these responses is unknown. Potential associations of racial discrimination with alterations in deep brain functional connectivity and accelerated epigenetic aging, a process that substantially increases vulnerability to health problems, are also unknown.

**Objective:**

To examine associations of racial discrimination with brainstem and midbrain resting-state functional connectivity (RSFC) and DNA methylation age acceleration (DMAA) among Black women in the US.

**Design, Setting, and Participants:**

This cohort study was conducted between January 1, 2012, and February 28, 2015, and included a community-based sample of Black women (aged ≥18 years) recruited as part of the Grady Trauma Project. Self-reported racial discrimination was examined in association with seed-to-voxel brain connectivity, including the locus coeruleus (LC), periaqueductal gray (PAG), and superior colliculus (SC); an index of DMAA (Horvath clock) was also evaluated. Posttraumatic stress disorder (PTSD), trauma exposure, and age were used as covariates in statistical models to isolate racial discrimination–related variance. Data analysis was conducted between January 10 and October 30, 2023.

**Exposure:**

Varying levels of racial discrimination exposure, other trauma exposure, and posttraumatic stress disorder (PTSD).

**Main Outcomes and Measures:**

Racial discrimination frequency was assessed with the Experiences of Discrimination Scale, other trauma exposure was evaluated with the Traumatic Events Inventory, and current PTSD was evaluated with the PTSD Symptom Scale. Seed-to-voxel functional connectivity analyses were conducted with LC, PAG, and SC seeds. To assess DMAA, the Methylation EPIC BeadChip assay (Illumina) was conducted with whole-blood samples from a subset of 49 participants.

**Results:**

This study included 90 Black women, with a mean (SD) age of 38.5 (11.3) years. Greater racial discrimination was associated with greater left LC RSFC to the bilateral precuneus (a region within the default mode network implicated in rumination and reliving of past events; cluster size *k* = 228; *t*_85_ = 4.78; *P* < .001, false discovery rate-corrected). Significant indirect effects were observed for the left LC-precuneus RSFC on the association between racial discrimination and DMAA (β [SE] = 0.45 [0.16]; 95% CI, 0.12-0.77).

**Conclusions and Relevance:**

In this study, more frequent racial discrimination was associated with proportionately greater RSFC of the LC to the precuneus, and these connectivity alterations were associated with DMAA. These findings suggest that racial discrimination contributes to accelerated biological aging via altered connectivity between the LC and default mode network, increasing vulnerability for brain health problems.

## Key Points

**Question:**

Is racial discrimination associated with brain connectivity, and are alterations in deep brain functional connectivity associated with accelerated epigenetic aging?

**Findings:**

In this cohort study of 90 Black women in the US, higher self-reported racial discrimination was associated with greater resting-state functional connectivity (RSFC) between the locus coeruleus (LC) and precuneus. Significant indirect effects were observed for the association between racial discrimination frequency and DNA methylation age acceleration.

**Meaning:**

These findings suggest that racial discrimination is associated with greater connectivity in pathways involved with rumination, which may increase vulnerability to stress-related disorders and neurodegenerative disease via epigenetic age acceleration.

## Teaser

This cohort study uses neuroimaging to examine the association of racial discrimination with alterations in resting-state functional connectivity and accelerated epigenetic aging among Black women in the US.

## Introduction

Black individuals in the US experience race-related stressors, including systemic injustices and racial discrimination on a regular basis.^1,2,3^ Repeated exposure to racial discrimination has been associated with a greater incidence of brain health disorders.^4,5,6^ Frameworks of racial trauma suggest that racial discrimination enacts its effects via activation of stress-sensitive regulatory systems: namely, the sympathoadrenomedullary system and the hypothalamic-pituitary-adrenal axis.^5,7^ Activation of these systems can also lead to greater arousal and vigilance for future racist threats and to rumination as individuals relive past events.^5,8,9,10^ Racial discrimination frequency has been linked to a greater prevalence and increased severity of brain health problems, ranging from mental health disorders (eg, posttraumatic stress disorder [PTSD] and major depressive disorder^8,11,12,13,14,15^) to cognitive disruptions and dementia.^16,17^ Epidemiologic studies suggest that Black individuals have a 2-fold greater risk of Alzheimer dementia compared with White individuals; racial discrimination has been indicated as a contributing factor.^18,19^ Despite numerous studies highlighting the associations between racial discrimination exposure and negative brain health outcomes, few empirical studies have examined racial discrimination–related neurobiological mechanisms that may underlie these outcomes.

Emerging neuroimaging research has shown that racial discrimination affects brain function and structure, and racial discrimination may lead to a proportionately greater response and connectivity in brain networks involved with threat processing and emotion regulation.^20,21,22,23,24,25^ Racial discrimination frequency has been associated with greater resting-state functional connectivity (RSFC) between the amygdala (a brain region critical for detection of threat-related cues) and brain regions involved in the processing of threat signals, including the thalamus (sensory integration), visual cortex (visual attention),^20,21^ and insula (salience and interoceptive processing).^22^ Racial discrimination has also been linked to a proportionally greater response in prefrontal cortical regions, which engage in top-down regulatory control of threat response (rostral and ventromedial prefrontal cortices).^23,24,25^ One study revealed a putative biological mechanism underlying these neural alterations, indicating that greater immune system activation (assayed by C-reactive protein levels) moderated the association between racial discrimination frequency and ventromedial prefrontal cortex activation in response to threat cues.^25^

Racial discrimination has been associated with compromised structure of brain regions involved in threat processing and emotion regulation. Greater self-reported racial discrimination is associated with proportionally lower rostral cingulate cortex thickness^26^ and with decrements in the integrity of various prefrontal white matter tracts,^27,28,29^ with some of the strongest effects observed in prefrontal pathways (ie, anterior cingulum bundle and genu of the corpus callosum). Furthermore, a recent study found that degraded anatomic integrity of these specific pathways mediated the association between racial discrimination frequency and the overall burden of medical disorders.^30^ Together, these studies link greater racial discrimination frequency with increased function and diminished structure of threat regulation circuits, although the biological mechanisms of these neuroplastic alterations are largely unknown.

The aforementioned studies did not address the potential involvement of deeper brain regions that are critical to coordinating states of arousal and threat vigilance and, similarly, have relevance to the pathogenesis of disorders such as Alzheimer disease; this includes brainstem and midbrain regions such as the locus coeruleus (LC), periaqueductal gray (PAG), and superior colliculus (SC), all of which have been highlighted in trauma responses.^31,32,33^ The LC is a primary source of noradrenergic neurotransmission in the brain. The LC region has wide projections throughout the brain^34^ and plays a central role in orchestrating adaptive responses to threat. The PAG projects to cortical and subcortical structures to facilitate coordination of cardiovascular, respiratory, and motor responses associated with emotional arousal.^35,36,37,38^ The SC, a midbrain region, has been implicated in the visual detection of threatening stimuli and coordination of defensive behaviors (eg, fight or flight, freezing or tonic immobility, and emotional shut-down responses) through its tight connections with the PAG.^32,39,40,41,42^

Frameworks of racial trauma highlight arousal and vigilance, as well as high-effort emotion regulation, as critical cognitive mechanisms that may underpin the association between racial discrimination and negative health outcomes.^5,7^ That is, racial discrimination may lead to greater regulation of emotional response to racism-related threats and greater vigilance for future threats; over time, effort expended from these processes may contribute to the deterioration of body regulatory systems. However, little is known about the involvement of deep brain regions, which facilitate coordination of these processes, in racial discrimination–related responses. Furthermore, it is unclear how these neuroplastic changes affect biological mechanisms (eg, epigenetic alterations) that may enhance risk for brain health disorders. Several studies conducted in Black populations with high trauma exposure^43,44^ suggest that trauma- or stress-related genomic modifications in the form of DNA methylation contribute to phenotypic changes associated with PTSD, including alterations in structure and function in threat networks.^45,46,47,48^ DNA methylation from select epigenomic loci has been combined to form a weighted index (eg, Horvath or Hannum clock^49,50^) and used to predict advanced cellular aging, as greater clock values have been linked to age-related disorders including cardiovascular disease and early mortality.^51^ Given the evidence of race-related differences in accelerated aging and the fact that racial discrimination has been specifically linked to accelerated cellular aging in Black populations,^52,53,54^ racial discrimination may produce functional brain connectivity changes that, in turn, affect DNA methylation age acceleration (DMAA); to our knowledge, this has not been examined in extant research.

The goals of this study were as follows. First, we performed seed-to-voxel functional connectivity analyses to examine associations between racial discrimination and resting-state functional connectivity (RSFC) of deep brain regions involved with threat detection and response (ie, LC, PAG, and SC). Second, we examined whether these functional connectivity patterns mediate the association between racial discrimination and an index of DMAA (Horvath clock) in a sample of Black women in the US. Given that participants were recruited for PTSD studies and we were interested in isolating associations between racial discrimination and functional connectivity and DMAA, we controlled for variance associated with other trauma exposure symptoms, PTSD symptoms, and age in statistical models.

## Methods

### Participants

For this cohort study, we used data for Black women (aged ≥18 years) recruited as part of the Grady Trauma Project, a collection of studies investigating risk and resilience for trauma-related disorders in a largely Black population in the US. We selected Black women for this study because this group experiences disproportionately high rates of stress-related health problems and brain health problems.^55,56,57,58^ Participants were recruited in general medical clinics (obstetrics and gynecology, diabetes, and internal medicine) in a publicly funded hospital located in Atlanta, Georgia. Participants who met the criteria for trauma exposure according to Criterion A for PTSD in the *Diagnostic and Statistical Manual of Mental Disorders*, (Fourth Edition),^59^ were eligible for study participation and provided informed consent. They were scheduled for a magnetic resonance imaging (MRI) scan on a separate visit (participant eligibility is detailed further in the eMethods in Supplement 1). For sequencing of DNA methylation and other biomarkers, a subset of 49 participants also provided a blood sample on a visit before the MRI scan. Oversight and approval of this study was provided by the Emory University Institutional Review Board and the Grady Research Oversight Committee. Informed consent was obtained before participants engaged in study procedures. The study conformed to the principles of the Declaration of Helsinki^60^ and followed the Strengthening the Reporting of Observational Studies in Epidemiology (STROBE) reporting guideline.

### Clinical Assessments

Racial discrimination was measured using the Experiences of Discrimination (EOD) questionnaire, which quantifies the number of racially discriminatory experiences endorsed throughout an individual’s lifetime.^61^ The Traumatic Events Inventory (TEI) was administered to quantify the number of different types of traumas experienced during the individual’s lifetime (childhood and adult trauma).^62^ The PTSD Symptom Scale (PSS; score range, 0-51) was used to examine the presence and severity of current PTSD symptoms.^63^ Total TEI and PSS scores were used as covariates in statistical analyses. The clinical assessments are detailed further in the eMethods in Supplement 1.

### Blood Collection and DMAA Assay

Blood was collected in the morning at 10 am (±1 hour) after overnight fasting a mean 84 days before the scan or study visit. Whole blood was collected in an EDTA blood tube from an indwelling catheter and then buffy coat was isolated by centrifugation (at 1000 × *g* for 15 minutes at 4 °C) and stored at −80 °C until DNA extraction and EPIC BeadChip assay (Illumina) were conducted. Horvath DMAA was calculated using an online calculator, which is derived from taking the difference between epigenetically measured age and actual chronological age^49^ (quality control is detailed in the eMethods in Supplement 1). Proportions of CD8T cells, CD4T cells, natural killer cells, B cells, monocytes, and neutrophils were estimated using the robust partial correlation method in EpiDISH, version 2.8.0 (Bioconductor),^64^ using an Infinium Methylation EPIC (Illumina) array-specific reference panel and were used in sensitivity analyses, given their potential consequences on DNA methylation.^65^

### MRI Acquisition and Image Processing

Magnetic resonance imaging was conducted on either of 2 identical 3T scanners (MAGNETOM TIM-Trio; Siemens) at Emory University, using identical acquisition parameters (eMethods in Supplement 1). Functional MRI data preprocessing and quality assurance were done on the CONN toolbox, version 21.a (CONN),^66^ using their default pipeline, which included functional scan realignment, slice timing correction, coregistration to MPRAGE (magnetization-prepared rapid acquisition gradient echo), spatial normalization, and smoothing with a full-width half-maximum isotropic gaussian kernel filter of 8 mm. Functional and structural images were normalized to Montreal Neurological Institute space (MNI152); other preprocessing details are provided in the eMethods in Supplement 1.

### Statistical Analysis

**Figure 1.**
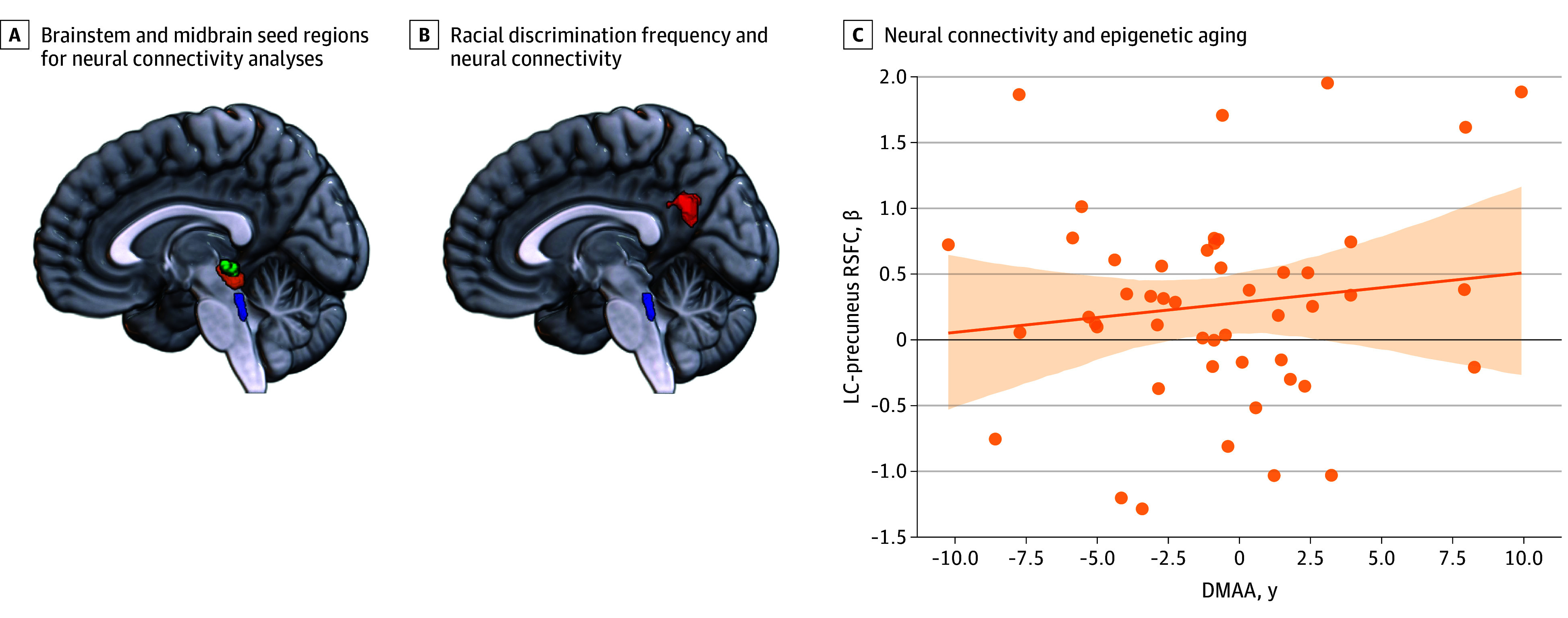
Association of Racial Discrimination With Brainstem Resting-State Functional Connectivity (RSFC) and Horvath DNA Methylation Age Acceleration (DMAA) A, Brainstem and midbrain seed regions for connectivity analyses: locus coeruleus (LC; blue), periaqueductal gray (orange), and superior colliculus (green). B, Frequency of racial discrimination is associated with greater RSFC between the LC (blue) and bilateral precuneus (red; Montreal Neurological Institute coordinates: *x* = −4, *y* = −60, and *z* = 24). C, Greater RSFC between the LC and bilateral precuneus is positively associated with Horvath DMAA (*r* = .30; *P* = .04). Each dot represents data from 1 participant. The shaded area represents the 95% CI, and the diagonal line represents the regression line.

Brainstem and midbrain seed regions for seed-to-voxel connectivity analyses were defined using the Brainstem Navigator toolkit,^67^ a collection of brainstem and diencephalic nuclei atlas labels and an MRI template.^68,69,70^ Seed-to-voxel generalized psychophysiological interaction analyses^71^ were conducted with the CONN toolbox to investigate associations between racial discrimination (EOD total score) and brainstem and midbrain RSFC (bilateral LC, PAG, and bilateral SC; Figure 1A) after covarying age, other trauma exposures (TEI total score), and current PTSD symptoms (PSS total score). Using the CONN toolbox, the threshold of connectivity significance was set at an uncorrected voxel-wise cluster-forming threshold of *P* < .001 (2-tailed) and a cluster significance threshold of *P* < .05 (2-tailed) with false discovery rate correction. Where significant results were observed, connectivity values were extracted from those regions and entered into mediational analyses, wherein the EOD total score was entered as an estimator; TEI total score, PSS total score, and age were entered as covariates; and Horvath DMAA values were entered as the outcome variable. Mediation and/or indirect effect analyses were conducted via the PROCESS macro, version 4,^72^ implemented in IBM SPSS, version 27 (IBM Corp). The PROCESS macro uses ordinary least-squares regression with bootstrapped CIs (5000 bootstrapped samples), which is well suited for mediation analyses with limited sample sizes.^73,74,75,76^ The threshold of statistical significance for mediation analyses was set at *P* < .05 (2-tailed). Data analysis was conducted between January 10 and October 30, 2023.

## Results

### Associations Between Racial Discrimination and Functional Connectivity

This study included a community-based sample of 90 women who self-identified as Black. Their mean (SD) age was 38.5 (11.3) years. Clinical and demographic characteristics of participants are described in the Table. No associations between racial discrimination and right LC RSFC were observed at our statistical threshold. However, greater racial discrimination was positively associated with left LC RSFC with the bilateral precuneus, even after adjusting for age, other trauma exposure, and PTSD symptoms (Montreal Neurological Institute coordinates: *x* = −4, *y* = −60, and *z* = 24; cluster size *k* = 228; *t*_85_ = 4.78; *P* < .001, false discovery rate corrected; Cohen *d* = 0.5; Figure 1B). No associations between racial discrimination and bilateral PAG or bilateral SC were observed.

**Table.** Participant Characteristics

Characteristic	Values^a^
Age, y	38.5 (11.3) [18-62]
Clinical assessment total score^b^	
EOD	2.4 (2.2) [0-8]
TEI	2.4 (1.4) [1-7]
PSS	11.4 (11.2) [0-50]
Education level^c^	
Less than 12th grade	12.2 (11)
High school graduate or GED	32.2 (29)
Some college or technical school	40 (36)
College graduate	11.1 (10)
Graduate school	3.3 (3)
Monthly income, $^d^	
≤249	11.1 (10)
250-499	11.1 (10)
500-999	32.2 (29)
1000-1999	25.6 (23)
≥2000	16.7 (15)

Abbreviation: GED, general educational development certification.

^a^ Values are presented as the mean (SD) with range in brackets or as the No. (%) of participants.

^b^ Racial discrimination exposure was assessed using the Experiences of Discrimination Questionnaire (EOD), other trauma exposure was evaluated with the Traumatic Events Inventory (TEI), and posttraumatic stress disorder was assessed with the PTSD Symptom Scale (PSS).

^c^ Missing 1 data point.

^d^ Missing 3 data points.

### Indirect Effect Analysis

**Figure 2.**
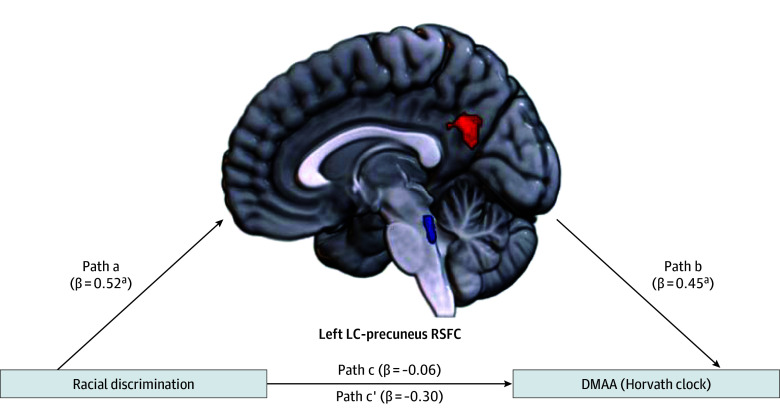
Association of Racial Discrimination With Horvath DNA Methylation Age Acceleration (DMAA) Through Left Locus Coeruleus (LC)-Precuneus Resting-State Functional Connectivity (RSFC) Significant indirect effects were observed (β [SE] = 0.45 [0.16]; 95% CI, 0.12-0.77). ^a^*P* < .05. ^b^*P* = .007.

Given that an association between racial discrimination and left LC-precuneus RSFC was observed, we extracted connectivity values from this cluster and entered it into a mediation analysis, with racial discrimination (EOD total) as the estimator, DMAA as the outcome variable, and left LC-precuneus RSFC as the mediator. Racial discrimination was a significant estimator of left LC-precuneus RSFC (path a: β = 0.52; *B* [SE] = 0.04 [0.01]; *P* < .001), and left LC-precuneus RSFC was a significant estimator of DMAA (path b: β = 0.45; *B* [SE] = 10.24 [3.6]; *P* = .007). Total racial discrimination was not a significant estimator of DMAA (path c: β = −0.06; *B* [SE] = −0.11 [0.27]; *P* = .67). However, the addition of left LC-precuneus RSFC made the overall model statistically significant (*F*_1,47_ = 4.13; *P* = .02; *R*^2^ = 0.15; Figure 2). Left LC-precuneus RSFC significantly mediated the association between racial discrimination and DMAA (β [SE] = 0.45 [0.16]; 95% CI, 0.12-0.77); however, the direct effect for the association between racial discrimination and age acceleration remained nonsignificant (path c’: β = −0.30; *B* [SE] = −0.57 [0.30]; *P* = .07; Figure 2). For sensitivity analysis, cell types (monocytes, natural killer cells, B cells, CD4T cells, and CD8T cells) were included as covariates in the mediation analysis (eResults in Supplement 1). In this model, a significant indirect effect of LC-precuneus RSFC was again observed for the association between racial discrimination and DMAA (β [SE] = 0.44 [0.17]; 95% CI, 0.11-0.80).

## Discussion

In this study, we examined the potential involvement of deep brain structures (LC, PAG, and SC) in racial discrimination–related RSFC alterations in a community-based sample of Black women in the US. We also tested for any potential indirect effects of seed-to-voxel functional connectivity on the association between racial discrimination and accelerated cellular aging, measured using an index of DMAA (Horvath clock). Our findings suggest that greater racial discrimination frequency was associated with greater RSFC between the left LC and the precuneus, a brain region implicated in rumination and reliving of past events, even after accounting for variance related to other types of trauma exposure, PTSD, or age. A statistically significant indirect effect was observed for LC-precuneus connectivity and the association between racial discrimination frequency and DMAA. Our data suggest that racial discrimination is associated with greater LC-precuneus connectivity and that these connectivity patterns are linked to greater cellular aging (possibly inclusive of brain aging) in Black individuals, amplifying the risk of brain health disparities.

Previous studies reported that greater experiences of racial discrimination are associated with greater RSFC between the left LC and precuneus, a key node within the default mode network—a system of brain structures that engage during idle states^77,78,79^ and are active during self-referential processing.^78,80,81^ The LC may modulate default mode network function via noradrenergic neurotransmission in component regions of the network. Investigators in one study observed that chemogenetic stimulation of tonic LC activity strengthened connectivity in this network.^82^ Greater default mode network activity may be a consequence of inadequate modulation of salience and executive control networks by the LC, biasing individuals toward nongoal-directed activity.^34,83^ Associations of racial discrimination with altered connectivity of the LC and a default mode network node highlight the potential importance of the LC in pathologic neurophysiologic adaptations to racial discrimination. This finding is meaningful not only in relation to default mode network function but also in the context of pathophysiologic models of neurodegenerative disease. Loss of LC noradrenergic neurons contributes to the pathophysiology of Alzheimer dementia,^84,85^ a disease that disproportionately affects Black individuals compared with White individuals and individuals of other ethnicities.^86,87^

Greater engagement and connectivity of default mode network regions (specifically, the precuneus) has also been associated with internalizing symptoms including anger, rumination, and reliving of traumatic memories.^88,89,90^ These processes are recognized as a common adaptation to repeated racial discrimination,^6^ whereby perseverative thoughts about discrimination-related events can enhance vigilance for similar potential events in the future as a strategy for avoidance of this threat. Internalizing symptoms have been previously linked to the acceleration of epigenetic aging in cross-sectional studies of children and adolescents.^91,92^ Notably, we found statistically significant indirect effects for the association of left LC-precuneus connectivity with racial discrimination and DMAA. Although speculative, this pattern of connectivity may reflect greater internalized emotion that could contribute to allostatic load (eg, the burden that stressors create on regulatory systems) and consequent epigenetic alterations that accelerate cellular aging. A 2021 study revealed that internalized, but not externalized, anger mediated the association between racial discrimination and accelerated epigenetic aging in Black women, suggesting that this may be a viable explanation for our findings.^93^ Relatedly, accelerated epigenetic aging has been associated with compromised white matter throughout the brain in different populations,^94,95^ and epigenetic age acceleration has been linked to stress-related disorders and neurodegenerative disorders.^45,46,47,48,96^ As such, our findings extend earlier research on accelerated aging in racially marginalized populations, showing links with brain mechanisms.^52,53,54^ They also bring forth important mechanistic insights into prior findings of greater gray and white matter decrements in relation to racial discrimination, highlighting the possibility that these associations could, in part, be due to epigenetic changes that cause accelerated tissue aging.^26,27,28,29,30^

### Limitations

We acknowledge several limitations of this study. Although we observed links among racial discrimination, altered functional connectivity in a network that supports rumination and internalized emotion, and accelerated aging, the study design precludes any assumptions of causality among these variables. Our connectivity seeds were brainstem and midbrain regions, thus limiting the scope of our analyses; similarly, a more extensive investigation of brain networks is needed to replicate our approach, which was conducted post hoc. Furthermore, we assessed the frequency of reported racial discrimination but not coping responses to these experiences. Given that we did not examine these psychological processes directly, our interpretations of associations between functional connectivity and engagement in these processes are speculative. As with other retrospective measures, there is the potential for recall bias affecting findings, and future studies with real-time assessment of racial discrimination are warranted to address these biases. Also, we assessed DNA methylation through assays conducted with peripheral blood. Although there is some evidence that methylation levels are conserved across different tissues, the data are inconsistent,^97^ and we cannot make conclusive statements that these peripheral assays of cellular aging are directly relevant to brain function or structure. Other socioenvironmental factors beyond our variables of interest affect DNA methylation, and further research with larger samples is needed to examine the unique contributions of these factors. We also acknowledge that the sample size for DNA methylation was limited, which reduced statistical power for performing more granular analyses with these data. Clearly, further research with larger sample sizes is needed to perform multivariate analyses with racial discrimination and other socioenvironmental variables. Additionally, only Black women were included in this study; as such, we could not examine potential sex differences in outcomes.

## Conclusions

 In this cohort study, we evaluated a potential neurobiological mechanism through which racial discrimination enhances vulnerability for brain health disparities in Black individuals. Our data highlight how racial discrimination may increase involvement of networks involved with rumination and internalization of emotion and suggest associations between racial discrimination–related neural connectivity alterations and accelerated cellular aging. We examined these associations in a sample of Black women, a group who experience disproportionately high rates of stress-related health problems and brain health problems.^55,56,57,58^ As such, the patterns of LC-precuneus RSFC observed in this study suggest a pathway through which racial discrimination can advance epigenetic aging and increase susceptibility to stress-related disorders and neurodegenerative disease.

## Notes

Supplement 1.
eMethods.

eResults.

eReferences

Supplement 2.
Data Sharing Statement
