# Supplementary material for: Racial Discrimination, Neural Connectivity, and Epigenetic Aging Among Black Women
Source: JAMA Netw Open. 2024 Jun 13;7(6):e2416588. doi: 10.1001/jamanetworkopen.2024.16588 (PMC11177169; doi:10.1001/jamanetworkopen.2024.16588)
Supplement: Supplement 1. — eMethods. eResults. eReferences [file jamanetwopen-e2416588-s001.pdf]

Supplementary Online Content

Elbasheir A, Katrinli S, Kearney BE, et al. Racial discrimination, neural connectivity, and epigenetic aging among Black women. *JAMA Netw Open*. 2024;7(6):e2416588. doi:10.1001/jamanetworkopen.2024.16588

eMethods.

eResults.

eReferences

This supplementary material has been provided by the authors to give readers additional information about their work.

© 2024 Elbasheir A et al. *JAMA Network Open.*

eMethods

Participant recruitment. Potential participants were screened with a questionnaire to assess for the presence of exclusion criteria which included: current usage of psychotropic medication, medical or physical conditions that would prevent MRI scanning (e.g., metal implants), any history of schizophrenia or other psychotic disorders, any medical conditions that might lead to onset of psychiatric symptoms (i.e., dementia), any history of head injury or loss of consciousness for longer than 5 minutes, or a history of neurological illness. After participants provided informed consent, clinical assessments related to racial discrimination, trauma history and PTSD symptoms were administered.

Clinical Assessments. The Experiences of Discrimination Questionnaire (EOD)(1) is a nine-item self-report measure of experiences of racial discrimination with good reliability and validity(1). Items query about discriminatory experiences due to race or ethnicity that occurred in various situations over their lifetime, including experiences in accessing housing, medical care, interactions with the police or government, or general interpersonal experiences. The following text prefaced questions about individual experiences: "Have you ever experienced discrimination, been prevented from doing something, or been hassled or made to feel inferior in any of the following situations because of your race, ethnicity, or color?" Participants were asked to endorse the number of different types of racially discriminatory experiences they had encountered. The Traumatic Events Inventory (TEI), developed in the context of the Grady Trauma Project (2), was administered to measure lifetime trauma exposure, inclusive of childhood and adult trauma; trauma types (number of types of trauma to which the person was exposed) was the TEI index included as a covariate in statistical analyses, calculated based on 15 possible types of trauma. The PTSD Symptom Scale (PSS(3)) a widely-used, reliable and valid measure of current PTSD symptoms (past two weeks), was also administered on the day of the MRI scan.

DNA Methylation Quality Control. Genome Studio was used to get raw methylation beta values. DNAm data quality control (QC) was done by R package *CpGassoc*. Samples with probe detection call rates <90% and average intensity values that were either less than 50% of the overall sample mean or below 2000 arbitrary units (AU) were removed. Probes with detection *p*-values >0.01 were set to missing. Probes that were missing in >10% of the samples and are cross-hybridizing were excluded (4).

© 2024 Elbasheir A et al. *JAMA Network Open.*

MRI acquisition parameters. All scanning was conducted on either of two identical research-dedicated Siemens 3 Tesla Siemens MAGNETOM TIM-Trio scanners (12-channel head coil): n=66 on one scanner and n=24 on the other scanner. High-resolution T1-weighted MPRAGE images were acquired with the following parameters: TE= 3.02 ms, TR= 2600 ms, flip angle= 8 degrees, matrix = 224 x 256, field of view = 224 x 256 mm, voxel size = 1x1x1 mm with sagittal slice orientation. Functional images (190 volumes) were acquired using a T2*-weighted gradient ZSAGA pulse sequence, which has been shown to recover susceptibility signal loss in both cortical and subcortical regions (30 interleaved transverse slices; 3.4 x 3.4 x 4.0 mm voxels; TR= 2950 msec; TE = 30/67 msec; flip angle = 90°; field of view = 220 x 220 mm) (5).

CONN toolbox Pre-processing. As part of CONN preprocessing steps, functional scans were subjected to motion outlier identification using the Artifact Detection Toolbox (https://www.nitrc.org/projects/artifact_detect/). The mean (SD) motion in our sample was 0.13mm (.06). RD (EOD total) was not significantly correlated with motion (r = -.06, *p* = .61) in our sample. Principal components filtering was used to identify anatomical noise (10 components for white matter, 5 components for cerebrospinal fluid); anatomical noise was included as a second-level covariate in statistical models.

eResults

Correlations between racial discrimination, age, PTSD symptoms and trauma exposure. Frequency of RD was significantly associated with PSS total score (r=.22; *p* = .03), age (r=.38; *p*=.001) and trauma exposure (TEI total; r=.36; *p* <.001).

Sensitivity Analysis of Indirect Effects. We repeated indirect effects analysis including cell types as covariates. In this model, the strength of the relationship between RD and left LC-precuneus rsFC was reduced and no longer significant (path a, β = .56, *B* = .05, SE *B* = .01, *p* = .07) although left LC-precuneus rsFC remained a significant predictor of DNAm age acceleration (path b, β = .41, *B* = 9.41, SE *B* = 3.68, *p* = .014). Total RD was not a significant predictor of age acceleration (path c, β =- .03, *B* = -.05, SE *B* = .29, *p* = .84). The addition of left LC-precuneus rsFC to the overall model was statistically significant, $F_{1,41}$ = 2.41, *p* = .037, R^2 = 0.29. Left LC-precuneus rsFC significantly mediated the relationship between RD and DNAm aging [β = 0.44, SE = 0.17, 95% CI (0.11, 0.80)], and the direct effect of RD on age acceleration remained non-significant (path c’, β =-.49, *B* = -.50, SE *B* = .32, *p* = .13).

© 2024 Elbasheir A et al. *JAMA Network Open.*

eReferences

1. Krieger N, Smith K, Naishadham D, Hartman C, Barbeau EM. Experiences of discrimination: Validity and reliability of a self-report measure for population health research on racism and health. Social Science & Medicine. 2005;61:1576-96.
2. Gillespie CF, Bradley B, Mercer K, Smith AK, Conneely K, Gapen M, et al. Trauma exposure and stress-related disorders in inner city primary care patients. General hospital psychiatry. 2009;31(6):505-14.
3. Foa EB, Riggs DS, Dancu CV, Rothbaum BO. Reliability and validity of a brief instrument for assessing post-traumatic stress disorder. Journal of traumatic stress. 1993;6:459-73.
4. McCartney DL, Walker RM, Morris SW, McIntosh AM, Porteous DJ, Evans KL. Identification of polymorphic and off-target probe binding sites on the Illumina Infinium MethylationEPIC BeadChip. Genom Data. 2016;9:22-4.
5. Heberlein KA, Hu X. Simultaneous acquisition of gradient-echo and asymmetric spin-echo for single-shot z-shim: Z-SAGA. Magnetic resonance in medicine. 2004;51(1):212-6.

© 2024 Elbasheir A et al. *JAMA Network Open.*
