# Supplementary material for: Racial Discrimination, Neural Connectivity, and Epigenetic Aging Among Black Women
Source: JAMA Netw Open. 2024 Jun 13;7(6):e2416588. doi: 10.1001/jamanetworkopen.2024.16588 (PMC11177169; doi:10.1001/jamanetworkopen.2024.16588)
Supplement: Supplement 2. — Data Sharing Statement [file jamanetwopen-e2416588-s002.pdf]

# Data Sharing Statement

Elbasheir. Racial Discrimination, Neural Connectivity, and Epigenetic Aging Among Black Women. *JAMA Netw Open*. Published June 13, 2024.
doi:10.1001/jamanetworkopen.2024.16588

**Data**
**Data available:** Yes
**Data types:** Deidentified participant data
**How to access data:** Requests for data can be sent to nfani@emory.edu
**When available:** With publication

**Supporting Documents**
**Document types:** None

**Additional Information**
**Who can access the data:** Researchers whose proposed use of the data has been approved
**Types of analyses:** For stated purpose in proposal
**Mechanisms of data availability:** with a signed data access agreement
